# Dr. Barry's "Code Sanitaire"

**Published:** 1831-01-01

**Authors:** 


A LETTER TO SIR JAMES M'GRIGOR, M.D., F.R.S., &c. ON THE SANITARY MANAGEMENT OF THE GIBRALTAR FEVER. By DAVID BARRY, M.D., &c.

THE author of the above letter conceives that a great saving of human life might be effected, in the event of another visitation of the fever at Gibraltar, by the timely issue of a well-digested code of sanitary regulations, based on the general and local history of the disease, commensurate with the actual state of science, and sanctioned by the constituted medical authorities of the country. He has therefore ventured to sketch out a *code sanitaire* of this kind, accompanied by a series of remarks on the nature, origin, &c. of the fever. These last we must pass over, in order to extract the sanitary code itself, which is short, and appears in most points judicious, whatever theory we may form of the nature or origin of the disease.

### CODE SANITAIRE.

"1. Let us never lose sight of the grand, inestimable facts, that the Gibraltar fever, in its former visitations, has rendered one portion of the population the invulnerable protectors of the other portion from its own future attacks, and that each individual, as he passes through its ordeal, is not only rendered safe himself, but becomes capable of being made a source of safety to many others.

2. When the disease shall have been proved to exist within the fortress, during the hot months, let the sick and the suspected be immediately removed without the walls, as nearly as possible in the manner practised in 1810, and there kept effectually separated from the healthy, unsuspected, susceptible part of the inhabitants.

3. Let the infected houses and goods be kept in strict quarantine, and purified by water, air, fumigations, and every other means that may be thought advisable; great care being taken that these expurgatory measures be executed by non-susceptible persons.

4. Let no time nor labour be thrown away, at this most important crisis, on cleansing drains or privies. Experience has already proved, most fully, both in Cadiz, in the great epidemic of 1800,* and in Gibraltar in 1828, the perfect inutility, nay, the absolute mischievous tendency of this measure, when adopted after the fever has commenced, with the view of arresting its propagation.

5. Should the infection appear to spread within the territory, notwithstanding the removal of the first sick, all theories must be abandoned, and one maxim must, alone, guide all our measures, viz. *that the disease will stop as soon as the susceptible are separated from contaminated places, persons, and things.*

6. Since, however, it would be obviously impracticable to remove all the susceptible from the fortress, at once, when an epidemic breaks out after a long interval of public health, and when besides a large portion of the civil population, the whole garrison may belong to this class, as was the case in 1828, we must send outside the walls all moveable foci and fomites of contagion, and as many as possible of those capable of being affected by such as cannot be removed.

7. The civil hospital, which stands nearly in the centre of the town, should be transferred, with its whole estab-

* Vide Vilalba (Epidemiologia Espanola), ann. 1800.

lishment, to the neutral ground, to serve as the nucleus of a civil lazaretto, on the very first breaking out of the disease. Regimental hospitals, also, should be sent out, as the corps to which they belong happen to be attacked.

8. No family, after having been once contaminated, should be allowed to remain an hour in the fortress, particularly at the commencement of an epidemic. Temporary emigration should be encouraged, amongst the civilians, by every possible means, and the whole susceptible population, civil and military, should be scattered over the neutral ground, the ships in the bay, Windmill hill, and Europa flat, as widely as the circumstances of the fortress, and the limited extent of the territory, will admit.*

9. Whenever a regiment becomes contaminated, it should be immediately encamped outside the fortress, if it can be spared: if it cannot, on Windmill hill, or Europa flat. There are no other situations within the walls, where the atmosphere is not close and sultry during the summer and autumn, and therefore improper for encampments.

10. The sanitary division of the healthy into the susceptible and the non-susceptible, naturally dictates the classification of the sick into the decided epidemic, the suspected, and the unsuspected. There should, therefore, be three distinct hospital establishments viz. 1. The foul lazaretto, for pronounced cases. 2. The lazaretto of observation, for those cases which may or may not turn out to be epidemic. 3. The free or clean hospital, for accidents and non-susceptible sick. All the attendants of the first and second establishments, medical, clerical, and others, should be kept, if possible, in quarantine.

* "It is much to be regretted, at least in a sanitary point of view, that a larger territory was not attached to Gibraltar, when its possession was secured to England by treaty. The high ground, about the Queen of Spain's Chair would be a most desirable situation for an epidemic encampment."

11. The bed, bedding, and every thing personal to the sick soldier, sent to either of the two first hospitals, should follow the fortunes of their owner. If the sick man should happen to die, his effects will thus remain where they can do no further mischief, viz. in the foul hospital: should he survive, they accompany him to the convalescent depôt, and thence, after having undergone the most careful ablutions, fumigations, &c. to the suspected quarter within the fortress, on his return to duty.

12. Hospital bedding, properly so called, should be used, as in time of public health, in the clean hospital only. This, of course, implies that the bed and bedding of the unsuspected sick need not be removed from the tents, or quarters of the healthy.

13. There should be three descriptions of camps and quarters, corresponding to the hospital establishment: the foul, the suspected, and the clean or free. These should be kept distinct during the epidemic.

14. Convalescents, from the foul and suspected hospitals, should be returned to the fortress, as soon as possible after their recovery, placed in suspected quarters, and appointed to the lightest duties at first, distinct from the uncontaminated, until the return of public health.

15. The guards, and all other duties within the town and in the sheltered situations of the territory, should be reduced to the minimum consistent with the safety of the fortress; and, as soon as the original and convalescent non-susceptible soldiers are sufficiently numerous to perform these duties, the susceptible should no longer be permitted to participate in them.

16. The building called the naval hospital, which, during the most fatal period of the late epidemic, was crowded with military sick, being constructed in the form of a hollow square, at the bottom of a close and deep ravine, should be abandoned as an hospital, on the very first breaking out of any future epidemic. It might, perhaps, be advantageously occupied as a station for nonsusceptible convalescents.

17. The epidemic sick should, as far as practicable, be treated in detached tents, huts, or sheds, so placed and constructed as to admit of the most perfect ventilation.

18. It will not be enough for the protection of the susceptible, nor for the benefit of the sick, that the latter be sent outside the gates, to the north front. They must be so placed as not to be sheltered by the projections of the rock, nor by the outworks, from the currents of cool air which constantly sweep round that face of the mountain, either from the Mediterranean to the bay, or in an opposite direction, whatever point the wind may blow from elsewhere. No spot, therefore, inside the line of the Orillon ditch and Bayside barrier, should be occupied as an epidemic hospital.

19. As the limits of the territory stand defined at present, on the land side, the most fitting situation for the epidemic sick, on the north front, would be the north-eastern angle of the neutral ground; for the suspected sick, a space near the former to the westward; for the unsuspected sick, still further to the westward: the three establishments to be placed in *echellon.*

20. Should it so happen that the troops in garrison cannot furnish a sufficient number of nonsusceptible orderlies for the service of their own epidemic sick, civil attendants of that class must be employed from the beginning.

21. In the pitching of tents, and particularly in the erection of boarded sheds, care must be taken that they be not huddled too closely together, and that they be so placed with regard to each other, as to allow a free passage for the currents of air mentioned in 18. Nothing tends more effectually to prevent the propagation of disease than open space and perfect ventilation.

22. As the town was evidently the centre, from which infection emanated during the first six weeks of the late epidemic, it would be advisable, on any similar occasion in future, immediately on the first appearance of the disease, to cut off all communication with the south, by shutting the gates on that side, and to preserve these two parts of the garrison perfectly distinct, at least as long as the people of the south might continue to afford no proofs of being infected. This measure I conceive to be easily practicable, and perfectly compatible with the safety of the fortress.

23. The first and most important steps towards the saving of human life, on the breaking out of this disease, being its early detection, and the firm, unhesitating announcement of its existence to the proper authority. The chief medical officer should, himself, visit and observe every case of febrile indisposition occurring withing the territory from the 15th of June to the 15th of November; and should see every dead body, and have it opened in his presence, if necessary, during that period."*

---

### LIII.

* Med. and Phys. Journal. December, 1830.

